# Artemether–lumefantrine treatment failure despite adequate lumefantrine day 7 concentration in a traveller with *Plasmodium falciparum* malaria after returning from Tanzania

**DOI:** 10.1186/1475-2875-11-176

**Published:** 2012-05-25

**Authors:** Anna Färnert, Johan Ursing, Thomas Tolfvenstam, Josea Rono, Lillemor Karlsson, Elda Sparrelid, Niklas Lindegårdh

**Affiliations:** 1 Infectious Diseases Unit, Department of Medicine Solna, Karolinska Institutet, Stockholm, Sweden; 2 Department of Infectious Diseases, Karolinska University Hospital, SE-171 76 Stockholm, Stockholm, Sweden; 3 Department of Clinical Microbiology, Karolinska University Hospital, Stockholm, Sweden; 4 Mahidol-Oxford Tropical Medicine Research Unit, Faculty of Tropical Medicine, Mahidol University, Bangkok, Thailand; 5 Centre for Tropical Medicine, Nuffield Department of Clinical Medicine, University of Oxford, Oxford, UK

**Keywords:** Malaria, *P. falciparum*, Artemisinin, Lumefantrine, Sensitivity, non-immune

## Abstract

Artemether-lumefantrine is currently first-line therapy of *Plasmodium falciparum* malaria in many countries. This report describes a treatment failure despite adequate drug concentrations in a traveller returning from sub-Saharan Africa. Genotyping confirmed recrudescence and suggested reduced sensitivity. Potential sub-optimal effect of artemether-lumefantrine highlights the need to follow non-immune individuals the weeks after treatment.

## Background

Artemisinin-based combination therapy (ACT) is recommended by the World Health Organization as first-line treatment of uncomplicated *Plasmodium falciparum* malaria in sub-Saharan Africa [1]. The combination comprises a fast-acting artemisinin derivative with rapid effect on parasite clearance, and a long-acting drug to prevent recrudescence and development of resistance. The fixed-dose oral combination artemether-lumefantrine (CoArtem®/Riamet®, Novartis, Switzerland) is increasingly used for treatment of *P. falciparum* malaria in travellers returning to non-endemic areas, and is the first-line drug for uncomplicated malaria at Karolinska University Hospital in Stockholm since 2010. The drug combination is also recommended as follow-up treatment to artesunate in patients recovering from severe *P. falciparum* malaria. The present report describes artemether-lumefantrine treatment failure in a traveller with *P. falciparum* malaria after returning from Tanzania.

## Case Report

A 57-year old man of Swedish descent presented at Karo-linska University Hospital, on June 29, 2011, with a one-day history of fever, chills, headache and loose stool, two weeks after returning from Tanzania where he had been working intermittently for a few years. He had discontinued atovaquone-proguanil chemoprophylaxis one month before leaving endemic area. He reported being previously healthy except an episode of necrotizing fasciitis in the left thigh six months earlier.

**Figure 1:**
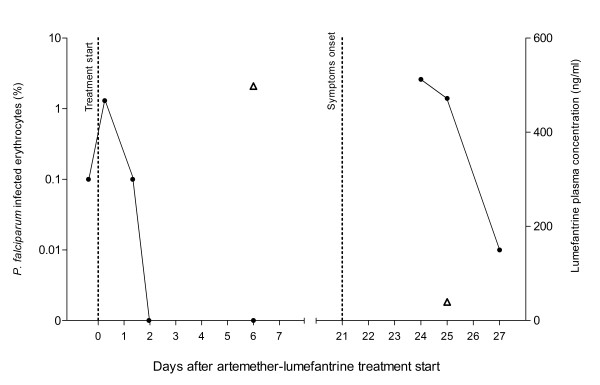
**Recrudescence of *P. falciparum* in a traveller treated with artemether-lumefantrine (Riamet®, Novartis).** The *P. falciparum* parasitaemia (% infected erythrocytes as filled circles) decreased promptly after artemether-lumefantrine treatment was initiated (day 0) and symptoms reappeared on day 21. The second episode was treated successfully with mefloquine. Plasma concentrations of artemether, dihydroartemisinin, lumefantrine and desbutyl lumefantrine were assessed on day 6 and day 25. Lumefantrine concentrations (open triangles) are shown in the graph whereas the other results are stated in the text.

On examination, the patient had prominent chills without any other clinical findings and C-reactive protein (CRP) was 166 mg/l, haemoglobin 171 g/l, platelet 72x10^9^/l, bilirubin 33 μmol/l. He was started on intravenous cefotaxime and fluids for a suspected bacteremia, and artemether-lumefantrine (20 mg/120 mg Riamet®; 4 tablets per dose at 0, 8, 24, 36, 48 and 60 hours) was prescribed after microscopy had shown 0.1% *P. falciparum* infected erythrocytes. The first dose was however delayed and parasitaemia peaked at 1.3% before decreasing promptly during treatment (Figure 1). Due to persistent fever and high CRP (369 mg/l) on the second day, cefotaxime was changed to ceftazidime and a single dose gentamicin for treatment of a potential concurrent bacteraemia. The patient was discharged free of symptom after five days and prescribed one week oral ciprofloxacin. Cultures from blood, urine and stool taken at and during admission were all negative. Malaria microscopy was negative at discharge as well as two days later.

On July 23 (24 days after treatment start), the patient presented at the emergency room with high fever since two-three days, jaundice, hypotension (systolic blood pressure 80) and slightly impaired consciousness. He responded promptly to intravenous fluids. CRP was 64 mg/l, hemoglobin 115 g/l, platelet count 116x10^9^/l, bilirubin 49 μmol/l, arterial blood gas and other tests were normal. Blood films were again positive for *P. falciparum* and oral mefloquine was started based on 0.4% parasitaemia estimated by the physician on call. The initial parasitaemia was reevaluated to 2.6% by the microbiology department the following day. The patient recovered promptly and the mefloquine course was completed. He was discharged three days after admission. On follow up one month later, the patient reported no symptoms since discharge and microscopy of blood films was negative for malaria parasites.

In order to elucidate the cause of treatment failure, frozen EDTA plasma and blood, sampled as part of a malaria immunology study, were assessed with the patient’s consent. Species-specific PCR of the ssRNA gene confirmed the presence of only *P. falciparum.* Genotyping of the polymorphic merozoite surface protein 2 gene (*msp2*) [2] detected the same two alleles (336 bp FC27 and 487 bp IC-1 fragments) at both episodes.

Drug concentrations were measured by liquid chromatography coupled to tandem mass-spectrometry (LC-MS/MS) in plasma collected on day 6 and 25 after initiating artemether-lumefantrine (Figure 1). Both artemether and dihydroartemisinin concentrations were below the limit of detection (0.5 ng/ml) in all samples as expected because of their very short half-lives (2–3 hrs) [3]. The lumefantrine concentrations were 498 ng/ml on day 6 and 39.5 ng/ml on day 25, while the metabolite desbutyl lumefantrine was 2.94 ng/ml and below detection (0.25 ng/ml), respectively.

Genotyping of drug resistance markers [4] revealed the same profiles at both episodes; the *P. falciparum* chloroquine resistance (*pfcrt)* 76 K allele, the multidrug resistance 1 (*pfmdr1)* 86 N allele, and single copies of the *pfmdr1* gene.

## Discussion

Artemether-lumefantrine is one of the most widely used ACT for treatment of uncomplicated *P. falciparum* malaria in sub-Saharan Africa. Previous four-dose regime was associated with 15% treatment failure, whereas, the now recommended six-dose regime showed, in a recent pooled analysis, a 28-day PCR-corrected parasitological cure rate of 97% in malaria endemic areas [5].

The effect of anti-malarial drugs in patients treated in non-endemic areas is less studied, but can be highly informative since patients are non-immune and not subject to re-exposure. Treatment failures with artemether-lumefantrine have been reported in two travellers after visiting Sierra Leone [6]and Congo [7]. Since lumefantrine is highly lipophilic and bioavailability depends on concurrent food intake [8], these failures have, in the absence of pharmacological assessments, been speculated to be due to sub-optimal lumefantrine concentrations. Also different sets of parasite populations have been suggested to have caused the recurrent episodes.

Here, a late treatment failure of artemether-lumefantrine is described in a traveller despite adequate plasma concentrations of lumefantrine. Genotyping showed the same *msp2, pfcrt* and *mdr1* patterns, confirming parasite recrudescence. Despite that the patient reported nausea (without vomiting) and not eating the first day, the lumefantrine concentration (498 ng/ml) on day 6 corresponded to what has been estimated to be sufficient to prevent recrudescence, i.e. day 7 concentration >280 ng/mL [8,9]. Artemether and dihydroartemisinin were, as expected due to their short half-lives, below the level of detection on day 6. A recent publication points to desbutyl lumefantrine possibly being a better predictor for treatment outcome than lumefantrine [10], and the desbutyl lumefantrine concentration in the traveller (2.96 ng/ml) was in the same but lower range of that in children with successful outcome (mean 15.5 range 0.6-58.2 ng/ml). Little is known about the pharmacokinetics of desbutyl lumefantrine and the exact relevance of a therapeutic threshold for the metabolite needs further studies.

Reduced *in vivo* sensitivity to artemisinin has so far only been reported from Cambodia as reduced parasite clearance time [11]. Reduced *in vitro* susceptibility to artemisinin has also been described in a traveller returning from South East Asia [12]. Here, the prompt reduction of parasitaemia suggests good effect of artemether/dihydroartemisinin. The late treatment failure might, however, suggest reduced sensitivity to lumefantrine since the concentrations were well above therapeutic cut-off. This is further supported by the finding of *pfcrt* 76 K and *pfmdr1* 86 N alleles that have been associated with a five-fold increase of lumefantrine inhibitory concentration *in vitro* (IC50) and also found to be selected for after treatment with artemether-lumefantrine *in vivo* in Kenya [13] and Tanzania [14]. Not finding multiple copies of *pfmdr1*, also associated with reduced sensitivity to lumefantrine, might argue against this. However, multiple copies of *pfmdr1* have only rarely been described in Africa. *In vitro* susceptibility testing could have confirmed resistance however parasites were not available for culture.

The patient was administered several antibiotics and paracetamol at the time of artemether-lumefantrine treatment. Some reduced effect (higher recrudescence rates) have been seen when co-medicated with quinine, a drug with the same biotransformation pathway as lumefantrine (CYP 3A4) [15]. Moreover, ciprofloxacin which has been shown to have anti-malarial effect [16] was administered for one week. Although the present co-medication is not expected to have affected concentrations, interactions with other drugs might need further investigations.

In the current, as well as previous treatment failures in travellers, symptoms reappeared on day 14–24 after starting artemether-lumefantrine, reflecting late clinical failure [6,7]. Interestingly, the symptoms were more pronounced and the parasite densities higher at recrudescence [7]. Although this is probably due to patients waiting longer before seeking care (not believing it to be malaria again), milder symptoms might have been expected considering potential “strain-specific” immunity.

Rapid reduction of parasite biomass and symptoms together with few side effects has forwarded artemether-lumefantrine as an attractive alternative to mefloquine and atovaquone-proguanil for treatment of uncomplicated *P. falciparum* malaria in travellers. Although several studies show that artemether-lumefantrine does not have full efficacy, the drug combination has meet WHO > 95% cure rates [1], and is recommended as six-dose regimen in all areas irrespective of levels of drug resistance or host immunity.

The treatment failure described here might have been due to reduced sensitivity to lumefantrine but suboptimal concentrations of desbutyl lumefantrine and a missed dose cannot be fully ruled out even if the lumefantrine concentrations were adequate. Drugs chosen for treatment of *P. falciparum* malaria, especially in non-immune highly vulnerable individuals such as children and travellers, should preferably be efficacious enough to allow for single missed doses or uneven drug absorption. In endemic areas, partial immunity is likely to contribute to the effect of antimalarial drugs and thus overestimate cure rates. In line with this, decreasing effect of artemether-lumefantrine was indeed seen in an area of decreasing malaria transmission and immunity [17].

The present case forwards the need to further monitor the effect of artemether-lumefantrine treatment of *P. falciparum* malaria. Patients should be well informed to seek prompt care in the event of fever the weeks after completing artemether-lumefantrine treatment.

## Conflicts of interest

All authors declare no conflicts.

## Authors’ contributions

AF, JU and NL conceived the case report. TT, LK and ES were involved in the management of the patient. JU and JR performed molecular typing. NL was responsible for analysis of drug concentrations. AF, TT, JU and NL wrote the paper. The authors read and approved the final manuscript.
